# Genes Identified by Visible Mutant Phenotypes Show Increased Bias toward One of Two Subgenomes of Maize

**DOI:** 10.1371/journal.pone.0017855

**Published:** 2011-03-10

**Authors:** James C. Schnable, Michael Freeling

**Affiliations:** Department of Plant and Microbial Biology, University of California, Berkeley, California, United States of America; St. Petersburg Pasteur Institute, Russian Federation

## Abstract

Not all genes are created equal. Despite being supported by sequence conservation and expression data, knockout homozygotes of many genes show no visible effects, at least under laboratory conditions. We have identified a set of maize (*Zea mays* L.) genes which have been the subject of a disproportionate share of publications recorded at MaizeGDB. We manually anchored these “classical” maize genes to gene models in the B73 reference genome, and identified syntenic orthologs in other grass genomes. In addition to proofing the most recent version 2 maize gene models, we show that a subset of these genes, those that were identified by morphological phenotype prior to cloning, are retained at syntenic locations throughout the grasses at much higher levels than the average expressed maize gene, and are preferentially found on the maize1 subgenome even with a duplicate copy is still retained on the opposite subgenome. Maize1 is the subgenome that experienced less gene loss following the whole genome duplication in maize lineage 5–12 million years ago and genes located on this subgenome tend to be expressed at higher levels in modern maize. Links to the web based software that supported our syntenic analyses in the grasses should empower further research and support teaching involving the history of maize genetic research. Our findings exemplify the concept of “grasses as a single genetic system,” where what is learned in one grass may be applied to another.

## Introduction

The grasses, the approximately 10,000 species in the family Poaceae, are one of the most ecologically and economically significant taxa on the planet. Comparative mapping of diverse grass species led to the conclusion that they are all similar in gene content and order [1], [2] to the point that it was argued grasses could be treated as a single genetic system, sharing map data, markers, and leveraging inter-specific hybrids to dissect the genes responsible for morphological variation between different grass lineages [3]. In other words, knowledge gained from the study of any one grass species could be quickly and directly applied to all other species in the family.

Among the grasses, maize is without question the species with the longest and most comprehensively documented history of genetic investigation. The rich genetic resources found in maize are the result of over a century of genetic investigation beginning with R. A. Emerson's small but distinguished group in the early 20^th^ century; see B. McClintock's unpublished note on this group [4]. The resulting set of characterized genes has the potential to be of great value in the genomics era and sets maize apart from many model systems of more recent origin. Until now the applications of this information in a genomic context have been severely limited by the lack of reliable connections between the data produced by geneticists studying individual genes and the datasets produced by genomicists who generally work at the level of whole genomes.

**Figure 1:**
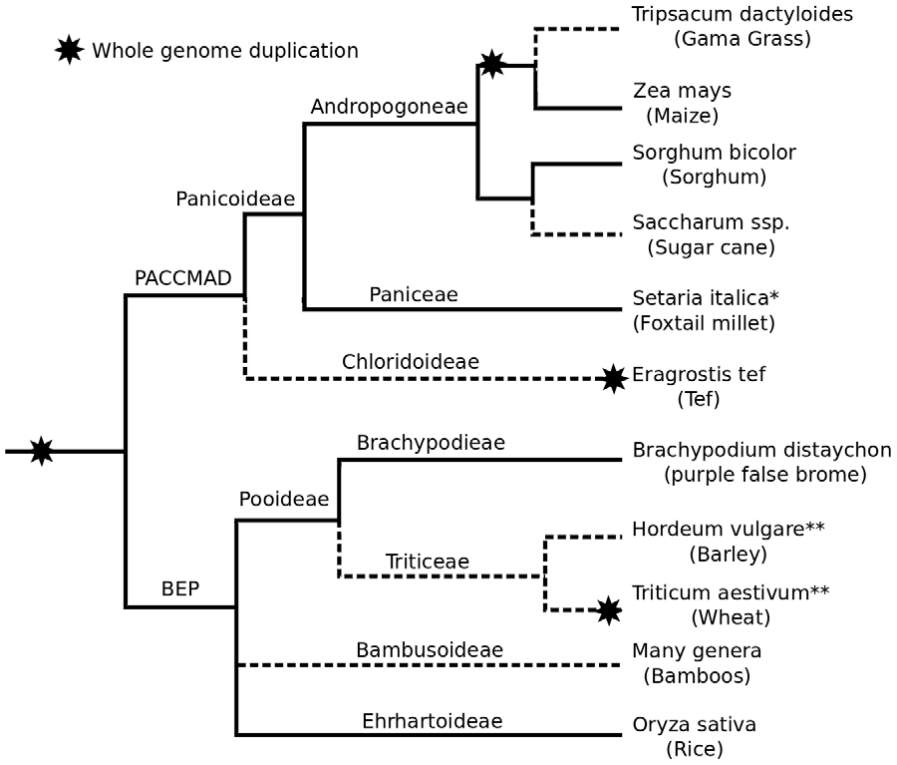
Phylogenetic relationships of notable and sequenced grass species. Branch lengths not to scale. *The genome sequencing of foxtail millet by the joint genome institute is complete, but has not yet been published. Therefore it is not included in our analyses (SI 1). **Projects to sequence the genomes of barley and wheat are announced or in progress.

We curated a dataset of 464 “classical” maize genes supported by citations from at least three publications, mutant phenotype data, or direct requests from the maize community using data presented in MaizeGDB: The Maize Genetics and Genomics Database (http://www.maizegdb.org) [5], [6]. Using manual annotation we connected these well characterized maize loci to gene models created by maizesequence.org, the group that recently published a sequence of the maize genome. To increase the utility of this dataset we also identified orthologous genes at syntenic locations in the genomes of three other grass species with published genomes: rice [7], sorghum [8], and brachypodium [9]. The evolutionary relationships of these grass species and a number of other notable grasses are shown in Figure 1. This initial classical gene list was distributed to the maize community with links to software that graphically presented our pan-grass synteny data and links to the MaizeGDB locus pages where all data regarding individual maize genes is archived.

The maize lineage, a branch that included both *Zea* and *Tripsacum*, experienced a whole genome duplication an estimated 5–12 million years ago [10]–[12]. This duplication created two homeologs (syn. homoeologs, ohnologs, syntenic paralogs) co-orthologous to single copy genes in other, unduplicated, grass species. The nearest unduplicated outgroup species with a sequenced genome is *Sorghum bicolor*. For many genes, the two duplicated copies were functionally redundant and one copy or the other has been lost from the genome of modern maize by an intrachromasomal recombination deletion mechanism [13]. Pairs of chromosomes orthologous to each of the ten chromosomes of sorghum can be reconstructed within the maize genome [14]. In all ten cases, one chromosome copy in maize has lost a significantly greater proportion of genes conserved syntenically in rice and sorghum across its entire length, and these chromosome copies are grouped together into the maize2 subgenome, while the chromosome copies that experienced lower rates of post-tetraploidy gene loss are grouped together into the maize1 subgenome [15].

Here we show that the genes of interest to maize geneticists are much more likely to be syntenically conserved across all grasses than the average gene supported by full length cDNA evidence. We also found that maize genes identified by a mutant phenotype are disproportionately found on maize1. The bias is true both for genes with a retained duplicate from the whole genome duplication, and singletons whose duplicate copies have been deleted. This finding was predicted by our previously published hypothesis that deletions of duplicate gene copies from the maize1 subgenome are more likely to impact fitness than deletions of copies of the same genes from maize2, as maize1 genes tend to be expressed at higher levels than their duplicates on maize2 [15]. We provide all our data on gene locus to gene model mapping, and identification of orthologous genes in other grasses and the homeologous gene in maize, if present, locations in the hopes that these data will be of use to others in the research and teaching community (Supplemental Information S1).

## Results

### Comparing gene models of individually cloned genes to gene models released by the maize genome sequencing consortium

**Figure 2:**
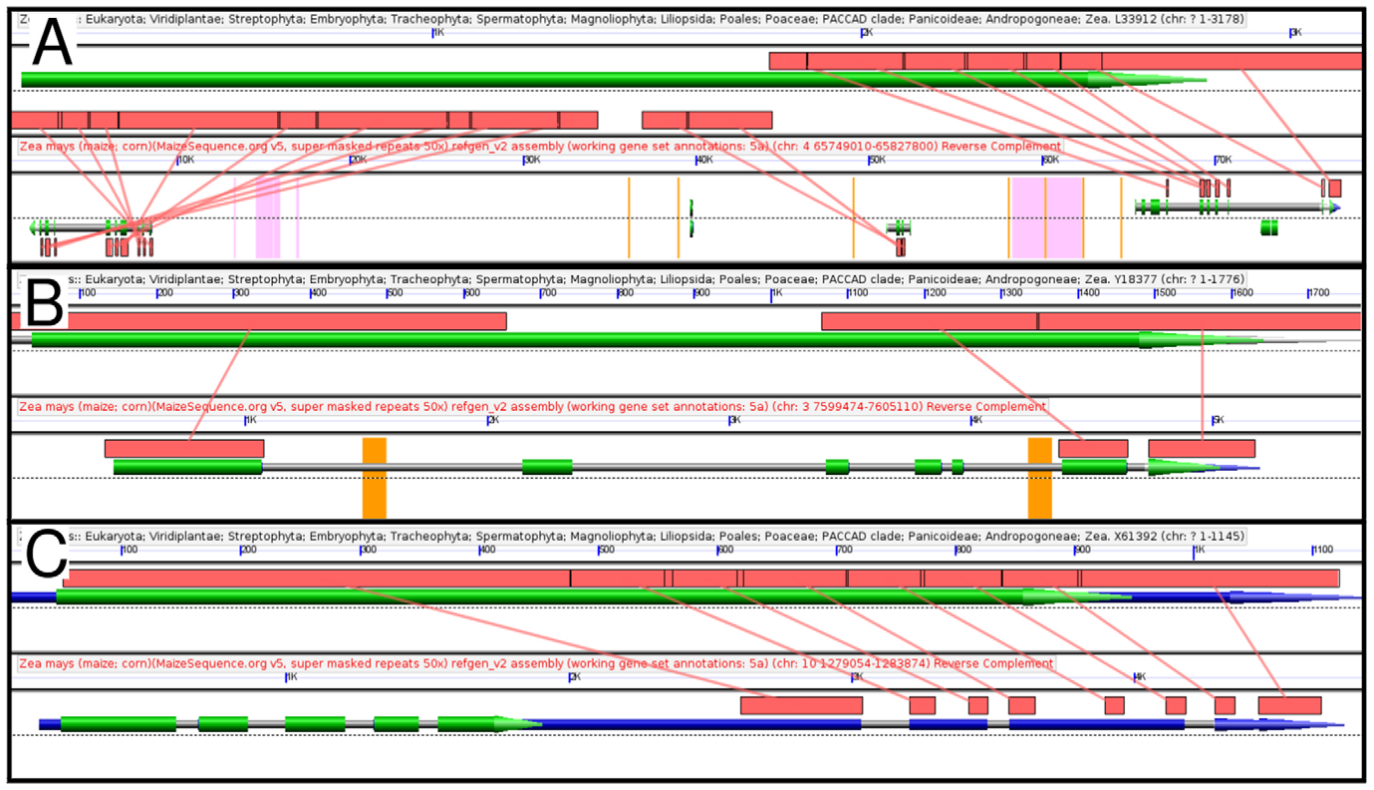
Examples of manually identified errors in maize gene annotations. Graphics from GEvo comparative sequence alignment tool. Annotated cDNAs from GenBank are compared to regions of the maize B73_refgen2 genome. Features on the forward strand are displayed above the dotted line, and features on the reserve strand are displayed below the line. Grey lines mark the extent of gene models with CDS sequences in green and UTR sequences in blue. Orange bars mark the gaps between assembled contigs of the maize genome (stretches of N's). Red boxes connected by lines show sequences identified as homologous by blastn. A. A comparison of the coding sequence of *aspartate kinase-homoserine dehydrogenase1* to the region of maize chromosome 4 that contains the three gene models –from left to right, GRMZM2G365423, GRMZM2G389303, and GRMZM2G437977 -- among which the exons of this gene have been divided. An interactive version of this graphic can be regenerated in GEvo using the following link: http://genomevolution.org/r/25xh B. A comparison of *cytokinin oxidase1* to GRMZM2G146644, a gene model which includes the 5' and 3' ends of *cko1* but has also incorporated unrelated exons from another maize genome contig. Regenerate analysis: http://genomevolution.org/r/25s5 C. The coding sequence of ferredoxin homeolog2 which maps to a region of the maize genome annotated as the 3' UTR of GRMZM2G147266. Regenerate analysis: http://genomevolution.org/r/25s7.

Manual mapping of experimentally validated genes to the maize genome provided a chance to error-check the version_2 gene models released by maizesequence.org. Overall most gene models agreed with previously cloned gene model data (Supplemental Information S1). Aside from missed UTR exons and the genes which were classified as supported only by *ab initio* prediction despite being supported by sequences in GenBank, the most frequent error we identified were genes that had been split into multiple unlinked gene models by maizesequence.org. This generally resulted from apparent mistakes in the ordering of contigs within BACs. The overall error rate was substantially reduced in the B73_refgen2 release, which increased the percent of contigs with order and orientation information from 30 to 80% [16]. However this form of error remains present in version 2. For example the coding sequence of the gene *aspartate kinase-homoserine dehydrogenase1* is split into three separate gene models (Figure 2A).

The most dramatic example of an erroneous gene model is provided by *cytokinin oxidase1*, where the 5' and 3' regions of the coding sequence mapped to the same gene model – GRMZM2G146644 – but the gene model included apparently unrelated exons from a contig inserted between the two ends of *cytokinin oxidase1* (Fig. 2B). In an additional two cases – *male sterile45* and *ferritin homolog2* -- the entire CDS of a gene mapped to regions annotated as UTR (Figure 2C). We provide proofing links in our master classical maize gene list so that a researcher can immediately visualize obvious annotation problems using the GEvo comparative genomics tool (a CoGe application) used to generate Figure 2 (Supplemental Information S1) [17].

### Comparing human to computational identification of maize genes using known sequences

Subsequent to the February, 2010 release of our initial version of classical maize gene list to the maize genetics community, maizesequence.org released a list of gene models mapped to named loci in the MaizeGDB database using the Xref computational pipeline (http://www.maizesequence.org/info/docs/namedgenes.html). Comparing their machine-annotated dataset to our version 2 list, we identified 152 cases of overlapping assignment of classical maize genes and named maize genes (Supplemental Information S1). The remaining 316 classical maize genes identified by manual annotation were not caught by the computational pipeline. In 140 of the overlapping cases, both lists assigned loci to the same gene model. The remaining 12 cases were further investigated using multiple independent GenBank records, as well as genetic location data recorded on MaizeGDB locus pages. In two cases the Xref assignment was clearly correct and the appropriate corrections were made to our list. In nine cases sequence and genetic location data supported the manual assignment over that of Xref. No conclusion could be reached in the final case.

### Identification of orthologs of classical maize genes in other grasses

The current release of the maize genome – B73_refgen2 – contains over 110,000 annotated genes, many of which have already been identified as gene fragments or genes encoding transposon related proteins. To develop a subset of genes comparable to our classical gene list we adopted an approach used previously [18] restricting ourselves to the subset of annotated maize genes supported by sequenced full length cDNA evidence (see Methods) [19], [20]. In total we identified 34,579 genes supported by full length cDNAs including 81.9% of the unique genes on our classical maize gene list and 75% of the unique genes which were originally identified by a visible mutant phenotype.

**Figure 3:**
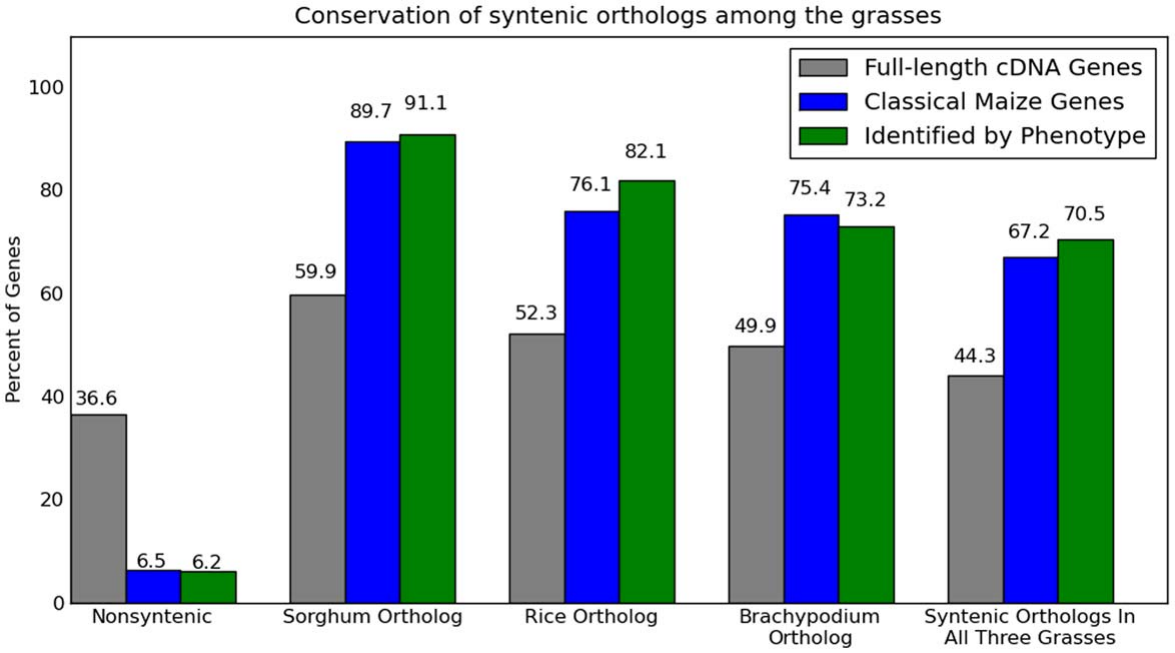
Syntenic conservation of the classical maize genes in other grasses. Comparison of the proportion of genes identified by a mutant phenotype prior to cloning (N = 111), all classical maize genes (N = 464), and all maize genes supported by full length cDNA evidence (N = 34579) for which syntenic orthologs could be identified in the other three grass species with sequenced genomes: sorghum, rice, and brachypodium.

Using the online syntenic analysis tool SynMap [21], we found that, compared to the average maize gene supported by full length cDNA evidence, classical maize genes, including those with known mutant phenotypes, are much more likely to possess conserved homologs at orthologous syntenic locations – true orthologs -- in *Japonica* rice, sorghum, and brachypodium (Figure 3).

### Distribution of classical maize genes and mutant phenotype genes between subgenomes

The maize genome is comprised of two subgenomes maize1 and maize2 [15]. Each subgenome is orthologous to the entire genomes of sorghum, rice, and brachypodium. These other grass genomes have remained unduplicated since the radiation of the grasses. The two subgenomes are distinguished by expression of retained duplicate genes and gene loss rates. Maize1 genes tend to be expressed at higher levels than their retained homeologs on maize2, and maize2 has lost copies of more genes syntenically retained in other grass species than maize1 [15].

**Figure 4:**
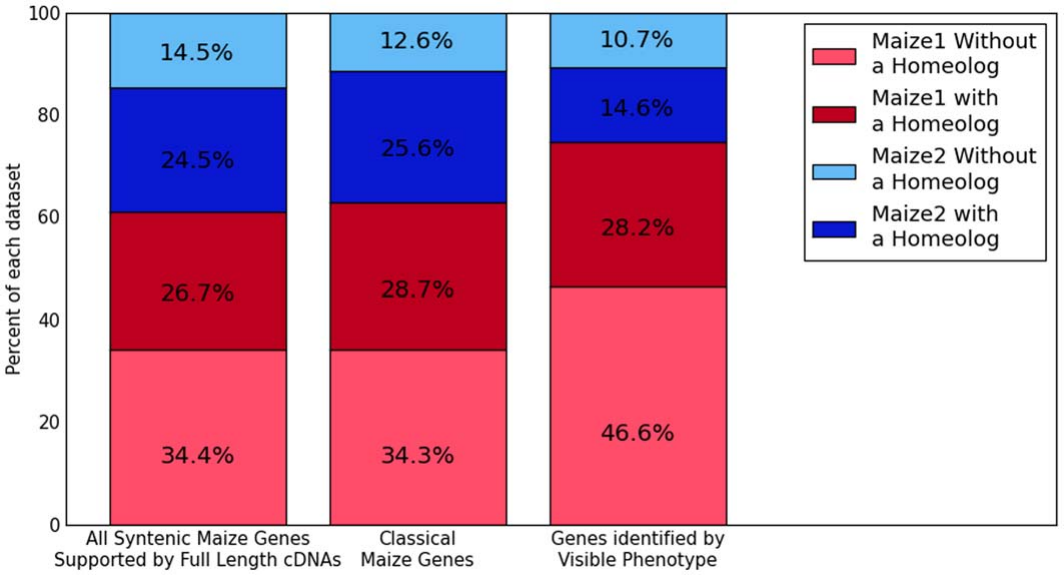
Distribution of classical maize genes between the two maize subgenomes. Comparison of the distribution of genes retained syntenically in at least one other grass species between the two subgenomes of maize as well as whether genes possess retained homeologs from the maize whole genome duplication. For syntenically retained maize genes with full length cDNA support N = 17956. For the subset of the classical maize gene list that are syntenically retained N = 429. For the subset of genes that were first identified by mutant phenotype and are syntenically retained N = 102.

The distribution of syntenically retained classical maize genes between the two subgenomes of maize roughly mirrors that of all syntenically retained genes supported by full length cDNA evidence. Figure 4 plots these data for all 34,579 genes supported by full length cDNA evidence, the 468 genes of the classical gene list, and the subset of 102 genes on the classical gene list identified by mutant phenotype prior to cloning. Given the bias towards greater expression of maize1 homeologs, the slight bias towards higher numbers of maize1 genes with retained homeologs among genes supported by full length cDNA evidence was expected, but this finding is not of significant interest. However, among syntenically retained genes which were first identified by a visible mutant phenotype, the bias towards the maize1 subgenome is significantly greater than for the classical maize gene list as a whole (p = .028, Fisher Exact Test), and members of homeologous gene pairs located on maize1 were twice as likely as the duplicate copies on maize2 to be originally identified by mutant phenotype -- 29 maize1 genes with homeologs vs. 14 maize2 genes with homeologs (significantly different from a 50/50 split p = .0222, Chi-square test).

## Discussion

### The benefits of manual gene annotation

Our manual proofing of the classical maize gene list shows that, as tempting as it may be to rely primarily on inexpensive *in silico* annotation techniques, manual structural annotation provided a significant amount of important information to B73_refgen2. Tools are available that allow interested researchers to proof and improve the structural annotations of their favorite genes [22]. Having those improvements incorporated into official genome annotations would benefit the entire community.

### Syntenic conservation of classical maize genes

The idea that genetic collinearity among the grasses could be used to accelerate the research across the whole family is a venerable one [1], [2], [23]. Enthusiasm for this concept of treating the grasses as a single genetic system waned as the sequencing of multiple grass genomes demonstrated that a significant fraction of transcribed genes are not syntenically retained across species, limiting the benefits of cross-species mapping and trait dissection. Our finding that 37% of maize genes supported by full-length cDNA are not retained at a syntenic position in other grass species, and almost 50% of cDNA supported genes apparently inserted into their present locations prior to divergence of the BEP clade, represented by both rice and brachypodium, is in agreement with previous studies. Research in arabidopsis, using papaya as an outgroup, estimated that half of all annotated genes in that species belonged to a “gray” genome of genes which had transposed into nonsyntenic positions within the last 70 million years [24]. A recent study in Drosophila found that knockouts of recently inserted – within the last 35 million years – and ancient syntenically conserved genes produced lethal phenotypes at statistically similar rates [25].

Genes belonging to the gray genome of maize are essentially unexplored. The genes of greatest interest historically seem to be precisely those that are retained in the same syntenic position in the genomes of all grass species. It may be that, in plants, genes essential for day to day function, such as those involved in key biochemical and developmental pathways, are by definition less likely to transpose or, when they transpose, are less likely to rise to fixation within a species. A small but significant number of mutant genes in maize were identified using map-based cloning approaches relying on rice synteny, prior to the publication of the maize genome. While map-based cloning and comparison of maize to rice certainly did occur, we think it unlikely that this explanation accounts for the magnitude of our results.

The techniques used in this paper allowed us to identify with high confidence, lost or transposed genes by first identifying a predicted orthologous syntenic location in the target grass genome. Even the genes which are not retained in all species can be a starting point for hypothesis driven research, a use we support via Gevo links to enable quick visual comparisons of orthologs or predicted locations in multiple grass species (Supplemental Information S1). For example, c1 and pl1 are two homeologous maize genes that regulate the biosynthesis of anthocyanin. Both genes have been studied extensively by the maize genetics community. A syntenic co-ortholog of the two genes is retained in the genomes of both sorghum and rice. However the gene is absent from orthologous region of the brachypodium genome (Figure S1) which prompted us to investigate further and find the gene was not present anywhere in the brachypodium genome (Figure S2). We conclude from this brief research foray that this portion of the anthocyanin biosynthetic regulatory pathway may be significantly different or completely absent in brachypodium, opening avenues for further research.

### Increased bias towards the maize1 subgenome of mutant phenotype genes

A bias towards maize1 for the classical maize genes was expected given the greater total number of retained genes present in that subgenome. However, when we examined the subset of the classical maize gene list identified by a mutant phenotype prior to cloning, the bias of this dataset towards the dominant subgenome – maize1 – was significantly greater than could be explained by the difference in total gene numbers between the two subgenomes. Interestingly this bias is also statistically significant for genes with a retained homeolog on the opposite, homologous subgenome, maize2. Since there is one gene copy present in each subgenome for this class of gene, *a priori* evidence of gene function, the expectation was that mutations of either copy would be about equally likely to produce a mutant phenotype. This was not the case.

Rather, our finding that maize1 is the preferred location of genes with mutant phenotypes even when a homeologous duplicate is present suggests that the loss of maize1 copies may be more likely to result in visible impacts of the sort which might catch the eye of researchers, or farmers, in the field. As impacts on plant morphology visible to researchers are likely to have a pronounced impact on plant fitness, this finding is certainly consistent with our previously published hypothesis that the deletion of a gene from maize1 is more likely to be selected against than the deletion of the same gene from maize2 [15].

The corollary is even more interesting: knockout phenotypes do not appear to be behaving as if gene function was buffered by a duplicate copy of the same gene expressed in the same cells. For the moment, our working hypothesis is that maize1 gene copies have predominantly retained the ancestral function of the gene in the pre-duplication ancestor of maize, leaving maize2 copies free to potentially adopt new, or less essential functions. This prediction is fully testable on a gene-by-gene basis through investigation of the function of orthologous genes we identify in the closely related and unduplicated species sorghum.

### Conclusion

This pilot study demonstrates the usefulness of traditional genetics data in the genomics era, and the importance of model species like maize with long histories of genetic investigation. A large number of morphological mutants in maize remain uncloned. The ability to identify high confidence orthologs in all grass species with sequenced genomes combined with the unrivaled economic and ecological significance of the Poaceae means investigation of a gene or gene family in any one of these species can quickly benefit researchers working around the world to answer a wide range of questions in different grass species. We hope that the tools, datasets, and links provided here (Supplemental Information S1), as well as our preliminary findings, will support continued insights based on pan-grass comparative genetics.

## Materials and Methods

Classical maize genes were identified from the list of maize loci maintained by MaizeGDB [5], [6] and include genes with associated GenBank sequence records with greater than three referencing papers in the database, additional cloned genes with known mutant phenotypes, as well as genes added after soliciting community input. Genes were initially mapped to the sequenced maize genome using LASTZ, and then visually proofed and corrected using GEvo part of the CoGe comparative genomics platform (http://genomevolution.org/CoGe/) [17]. These GEvo links are provided to aid continued research and permit proofing and verification of our results.

The full length cDNA-supported gene set was constructed using the 'semi-strict assembly' collection of full length cDNAs provided by the maize cDNA project (http://www.maizecdna.org) [19]. Full-length cDNAs were aligned to B73_refgen2 gene models using LASTZ, and those models supported by a full length cDNA with >95% identity and >90% coverage were included in the set.

Homeologous genes in maizes and orthologous genes in other grasses were identified using SynMap [21] with the optional Quota Align filters; SynMap is a web based tool available at http://www.genomevolution.org/CoGe/SynMap.pl. When no syntenic gene was identified, a predicted location was generated based on syntenically conserved flanker genes. Predicted orthologous locations longer than 1 MB were excluded as were predicted homeologous locations in maize longer than 2 MB. Our classical maize gene list provides a GEvo link that permits quick visual comparisons among grass orthologs and the predicted locations of deleted grass genes.

## Supporting Information

Figure S1 **Absence of a gene homologous to c1/pl1 in the predicted orthologous location of brachypodium.** GEvo Graphic (see legend of Figure 2) showing the conservation of similar genes in the same positions up and downstream of the homeologous maize genes *colored alurone1* and *purple plant1.* The same flanking genes are found in the same positions relative to the single orthologous genes in the sorghum and rice genomes. The location of these same genes has been used to predict the location where an orthologous genes in brachypodium should be located, however no sequence – annotated as a gene or otherwise – homologous to c1/pl1 is present at the predicted location.
(TIF)

Figure S2 **The a maximum likelihood tree showing the phylogenetic relationships of** ***colored alurone1/purple plant1*** **-like genes in maize, sorghum, rice, and brachypodium.** Based on syntenic location, these genes are predicted to fall into three clades of orthologous genes marked in yellow, green, and purple. The two genes most similar to c1/pl1 in brachypodium both fall into separate gene clades based on both tree topology and syntenic location.
(TIF)

Supplemental Information S1 (XLS)
