# Supplementary figures and images for: Genes Identified by Visible Mutant Phenotypes Show Increased Bias toward One of Two Subgenomes of Maize
Source: PLoS One. 2011 Mar 10;6(3):e17855. doi: 10.1371/journal.pone.0017855 (PMC3053395; doi:10.1371/journal.pone.0017855)

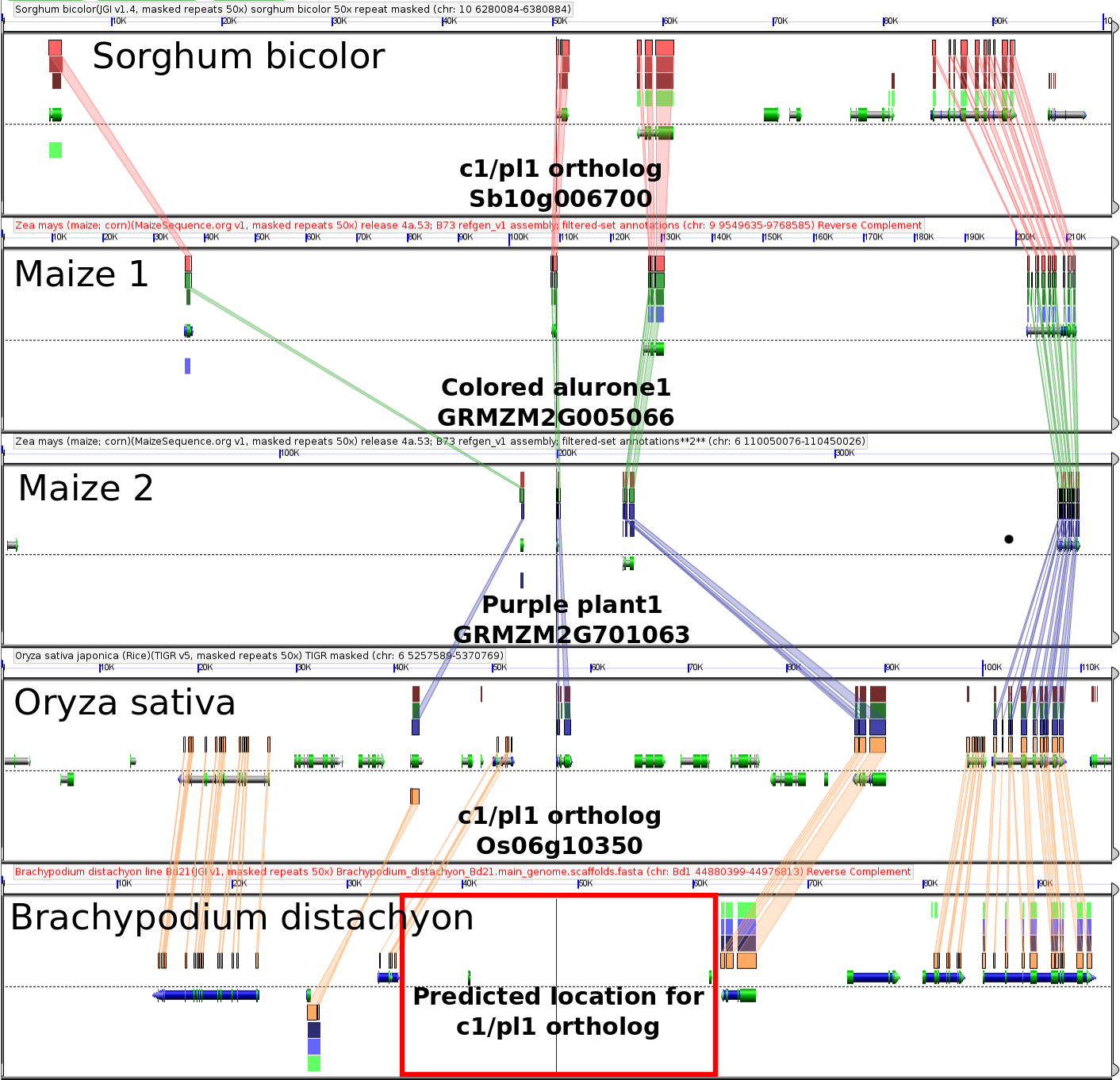

Supplement: Figure S1 — Absence of a gene homologous to c1/pl1 in the predicted orthologous location of brachypodium. GEvo Graphic (see legend of Figure 2) showing the conservation of similar genes in the same positions up and downstream of the homeologous maize genes colored alurone1 and purple plant1. The same flanking genes are found in the same positions relative to the single orthologous genes in the sorghum and rice genomes. The location of these same genes has been used to predict the location where an orthologous genes in brachypodium should be located, however no sequence – annotated as a gene or otherwise – homologous to c1/pl1 is present at the predicted location. (TIF) [file pone.0017855.s001.tif]

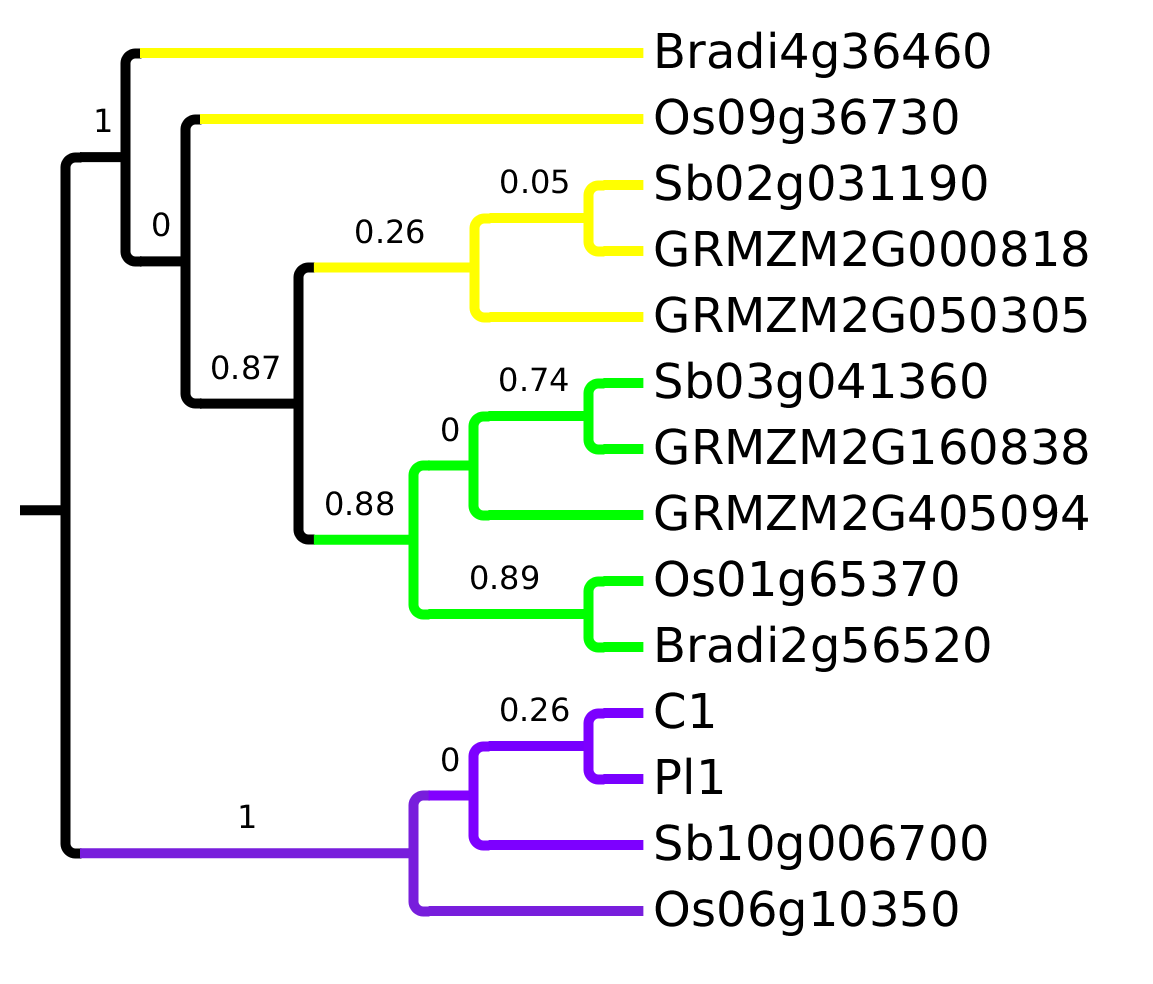

Supplement: Figure S2 — The a maximum likelihood tree showing the phylogenetic relationships of colored alurone1/purple plant1 -like genes in maize, sorghum, rice, and brachypodium. Based on syntenic location, these genes are predicted to fall into three clades of orthologous genes marked in yellow, green, and purple. The two genes most similar to c1/pl1 in brachypodium both fall into separate gene clades based on both tree topology and syntenic location. (TIF) [file pone.0017855.s002.tif]
